# An Important Admission

**Published:** 1886-04

**Authors:** 


### AN IMPORTANT ADMISSION.

We grow, the world grows and with it, at last, the medical faculty. Nay, the medical faculty have ever grown, but it has grown very slowly. Indeed, it assumed so much in its early existence, that it had to learn by degrees to know how little it really did know. How many graves have been filled by them; but "dead men tell no tales," so they are forever safe as to the past but are learning for the future.

When Dr. Meigs, of Philadelphia, is bold enough to come squarely up to the truth and state that "There are, however, many diseases even among those we are most commonly called upon to deal with, which are little, if at all, controlled in their course by any of the known drugs. Such diseases we can only treat by a well management of the diet, with stimulants if required, and regimen." Just think, a few years ago a physician would have been turned out of the profession as a quack, who should have undertaken to give the world such light. Medicine must be taken. That was the only theory. Don't allow water to be drank in fever; take your medicine and die according to rule. Now Dr. Meigs speaks and tells, to some degree, his experience:

"I am more and more struck at the end of each yearly hospital service with the fact that I lose very few of my typhoid fever patients who were brought to the hospital in the early stage of the disease. What is the treatment that effects results so much to be desired and so superior to those attained in many other diseases? It is attention to the following points—first, and most important, the diet; second, the enforcement of absolute rest; and third, the administration of medicine. By careful attention to these three indications for treatment, and by giving them prominence in the order in which they are named, comparatively few of those attacked will die, if only the treatment is instituted early, and they will be those in whom the "malignant influence" mentioned by Stokes, assumes from the beginning too preponderating a role for our supporting measures to overcome:'

"With regard to the permanent importance of careful attention to matters of diet in the treatment of digestive diseases of children, it is hardly necessary to say so much, especially as I have elsewhere treated of the subject. Beside, the fact that in diet do we possess our most powerful means for cure of this class of disease is so patent that it is almost universally acknowledged."

Now, this is all regular and of most excellent substance, but how many know that it has been argued and used by some doctors long ago, and rejected by the profession, until some such man as Meigs speaks out and tells his experience on the subject. The world moves.

